# The mitochondrial genome of *Oreogeton* sp. (Diptera: Empididae)

**DOI:** 10.1080/23802359.2019.1631129

**Published:** 2019-07-12

**Authors:** Qicheng Yang, Shang Gao, Zhaohui Pan, Ding Yang

**Affiliations:** a College of Food Science, Tibet Agriculture and Animal Husbandry University, Tibet, China;; b College of Plant Protection, China Agricultural University, Beijing, China

**Keywords:** Mitochondrial genome, Oreogetoninae, phylogenetics

## Abstract

The dance fly *Oreogeton* sp. belongs to the subfamily Oreogetoninae of Empididae. The mitogenome of *Oreogeton* sp. (GenBank accession number: MK639348) was sequenced, the first representative of the mitogenome of the subfamily. The nearly complete mitogenome is 15,718 bp totally, consisting of 13 protein-coding genes, two rRNAs, and 22 transfer RNAs. All genes have the similar locations and strands with that of other published species of Empididae. The nucleotide composition biases toward A and T, which together made up 77.2％of the entirety. Bayesian inference analysis strongly supported the monophyly of Empidoidea, Empididae, and Dolichopodidae. This result also suggested that Oreogetoninae was assigned to the sister group to the clade that consists of Trichopezinae and Clinocerinae, and then Empidinae is the sister to the clade that contains Oreogetoninae, Trichopezinae, and Clinocerinae.

## Introduction

Empididae is one of the largest families in Diptera with over 5000 described species from the world. Both adults and larvae of dance fly are predatory, which are the natural enemies for the pests that damage crops, fruiters, woods, and sanitation (Yang et al. 2007). They are widely used as a biological indicator of evaluating the quality of environment and biodiversity (Yang and Yang 2003).

The specimens of *Oreogeton* sp. used for this study were collected in Diebu County of Gansu by Shuangmei Ding and identified by Ding Yang. Specimens are deposited in the Entomological Museum of China Agricultural University (CAU). The total genomic DNA was extracted from the whole body (except head) of the specimen using the QIAamp DNA Blood Mini Kit (Qiagen, Germany) and stored at −20 °C until needed. The mitogenome was amplified and sequenced as described in our previous study (Wang et al. 2016). The nearly complete mitogenome of *Oreogeton* sp. (GenBank accession number: MK639348) is 15,718 bp. It encoded 13 PCGs, 22 tRNA genes, and two rRNA genes and the control region could not be sequenced entirely in this study and were similar with related reports before (Li et al. 2017; Zhou et al. 2017; Gao et al. 2018). All genes have the similar locations and strands with that of other published Empididae species. The nucleotide composition of the mitogenome was biased toward A and T, with 77.2% of A + T content (A = 39.8%, T = 37.4%, C = 13.6%, G = 9.3%). The A + T content of PCGs, tRNAs, and rRNAs is 75.0%, 77.3%, and 82.0% respectively. The total length of all 13 PCGs of *O.* sp. is 11,278 bp. Five PCGs (*NAD2*, *NAD3*, *ATP8*, *NAD5* and *NAD6*) initiated with ATT codons, and six PCGs (*COII*, *COIII*, *ATP6*, *NAD4*, *NAD4L*, and *CYTB*) initiated with ATG codons, *CO1* and *NAD1* initiated with TCG and ATA as a start codon, respectively. Twelve PCGs used typical termination codons TAA except *NAD5* used T in *Oreogeton* sp.

**Figure 1.**
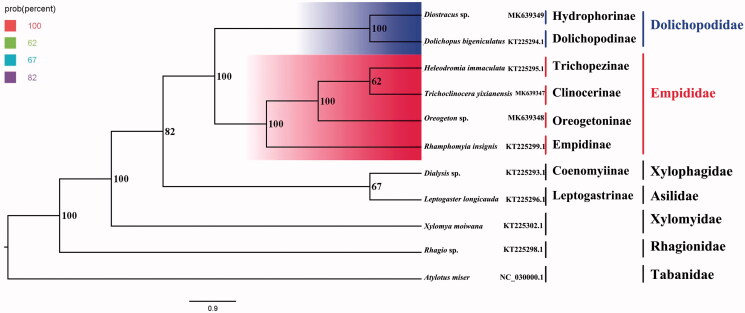
Bayesian phylogenetic tree of 11 Diptera species. The posterior probabilities are labeled at each node. Genbank accession numbers of all sequence used in the phylogenetic tree have been included in the Figure 1 and corresponding to the names of all species.

Phylogenetic analysis was performed based on the nucleotide sequences of 13 PCGs from 11 Diptera species. Bayesian (BI) analysis generated the phylogenetic tree topologies based on the PCGs matrices (Figure 1). According to the phylogenetic result, the monophyly of Empidoidea was strongly supported. The monophyletic Dolichopodidae, which contains Dolichopodinae and Hydrophorinae, was assigned to the sister group to the clade of monophyletic Empididae that consists of Empidinae, Clinocerinae, Oreogetoninae, and Trichopezinae in this study. For the phylogeny within Empididae, Clinocerinae is the sister to the clade of Trichopezinae, and the Oreogetoninae was assigned to the sister group to the clade that consists of Trichopezinae and Clinocerinae, and then Empidinae is the sister to the clade that contains Oreogetoninae, Trichopezinae, and Clinocerinae. This result that Dolichopodidae and Empididae are monophyletic respectively is consistent with the phylogenetic result of the previous research (Yang et al. 2007). The mitogenome of *Oreogeton* sp. could provide the important information for the further studies of Empidoidea phylogeny.
